# The Diabetes and Emotional Health Handbook and Toolkit for Health Professionals Supporting Adults With Type 1 and Type 2 Diabetes: Formative Evaluation

**DOI:** 10.2196/15007

**Published:** 2020-02-21

**Authors:** Jennifer A Halliday, Jane Speight, Andrea Bennet, Linda J Beeney, Christel Hendrieckx

**Affiliations:** 1 School of Psychology Deakin University Geelong Australia; 2 The Australian Centre for Behavioural Research in Diabetes Diabetes Victoria Melbourne Australia; 3 Applied Health Psychology Research Hornchurch, Essex United Kingdom; 4 Sydney Medical School The University of Sydney Sydney Australia; 5 Diabetes & Medical Psychology Services Normanhurst Australia

**Keywords:** diabetes mellitus, mental health, medical reference books, needs assessment, evaluation studies, qualitative research

## Abstract

**Background:**

Health professionals have expressed unmet needs, including lacking the skills, confidence, training, and resources needed to properly attend to the psychological needs of people with diabetes.

**Objective:**

Informed by needs assessments, this study aimed to develop practical, evidence-based resources to support health professionals to address the emotional needs of adults with type 1 or type 2 diabetes.

**Methods:**

We developed a new *handbook* and *toolkit* informed by formative evaluation, including literature reviews, stakeholder consultation and review, and a qualitative study. In the qualitative study, health professionals participated in interviews after reading sections of the *handbook* and *toolkit*.

**Results:**

The literature review uncovered that psychological problems are common among adults with diabetes, but health professionals lack resources to provide related support. We planned and drafted resources to fill this unmet need, guided by stakeholder consultation and an Expert Reference Group (ERG). Before finalizing the resources, we implemented feedback received from stakeholders (ERG, health professionals, academics, and people with diabetes). The resulting resources were the practical, evidence-based *Diabetes* and *Emotional Health* handbook and toolkit. A total of 19 health professionals took part in the qualitative study about the *handbook* and *toolkit*. They viewed the resources favorably, felt empowered to support people with diabetes experiencing psychological problems, and felt motivated to share the resources with others. Some gave examples of how they had used the *handbook* in clinical practice. A perceived highlight was the inclusion of a process model outlining 7 steps for identifying and supporting people with emotional problems: the 7 A’s model. With funding from the National Diabetes Services Scheme (NDSS), more than 2400 copies of *Diabetes and Emotional Health* have been distributed. It is freely available on the Web. The NDSS is an initiative of the Australian Government administered with the assistance of Diabetes Australia.

**Conclusions:**

The new evidence-based resources are perceived by stakeholders as effective aids to assist health professionals in providing emotional support to adults with diabetes. The 7 A’s model may have clinical utility for routine monitoring of other psychological and health-related problems, as part of person-centered clinical care.

## Introduction

### Diabetes and Emotional Health

Diabetes is a serious chronic condition affecting more than 415 million people worldwide, and this number is rising [1]. The 2 most common forms are type 1 and type 2 diabetes. Diabetes is characterized by high blood glucose levels, which over many years increase the risk of diabetes-related complications. Therefore, it is important to manage the condition to maintain optimal glucose levels (and minimize other risk factors) [2]. This is highly reliant on daily self-care (eg, taking medications, checking glucose levels, and maintaining a healthy lifestyle). Living with and managing diabetes can place considerable psychological burden on the person and their family [3]. Psychological problems can be both diabetes-specific (eg, diabetes distress and fear of hypoglycemia) and generic (eg, depression and anxiety) [4]. They are relatively common among adults with both types of diabetes, with many similarities in terms of the nature and prevalence of presenting problems as well as the strategies to address them. Preservation of psychological well-being is important in its own right [5] but impaired well-being is also associated with suboptimal diabetes self-care, biomedical outcomes (eg, HbA_1c_), and quality of life.

### Recommendations for Holistic Diabetes Care and Unmet Needs of Health Professionals

Clinical guidelines recommend person-centered, holistic diabetes care, including routine monitoring for psychological problems [6-9]. However, such guidelines are rarely implemented in clinical practice and the emotional health needs of people with diabetes often go unmet [10-13]. Few evidence-based, comprehensive, practical resources exist for health professionals about how to implement psychological care, beyond book chapters [14,15], books highly specific to 1 psychological problem [16,17], and student texts [18]. Although these have value, they are not freely available to health professionals and are limited in their practical application. For example, they are often heavily text-based or do not provide a step-by-step guide, including the practical elements that health professionals need, such as practice points, suggestions for open-ended questions and responses, and copies of validated screening tools for psychological problems. Health professionals have expressed unmet needs, including lacking skills, confidence, training, and resources, to properly attend to the psychological needs of people with diabetes [19-21]. Recommendations have been made recently for improved communications between health professionals and people with diabetes and for additional training and improved skills among health professionals to assist with overcoming these barriers [22].

### Aim and Objectives

Therefore, our aim was to address health professionals’ unmet needs, using a formative evaluation approach, by developing a practical, evidence-based resource: *Diabetes and Emotional Health: A handbook and toolkit for health professionals supporting adults with type 1 or type 2 diabetes* [23]. The objectives of the *handbook* and *toolkit* were to raise awareness, provide practice points and tools, and foster skill development, for identifying, communicating about, and addressing psychological problems experienced by adults with diabetes. This paper describes the formative evaluation of the *handbook* and *toolkit*.

## Methods

### Overview

**Figure 1:**
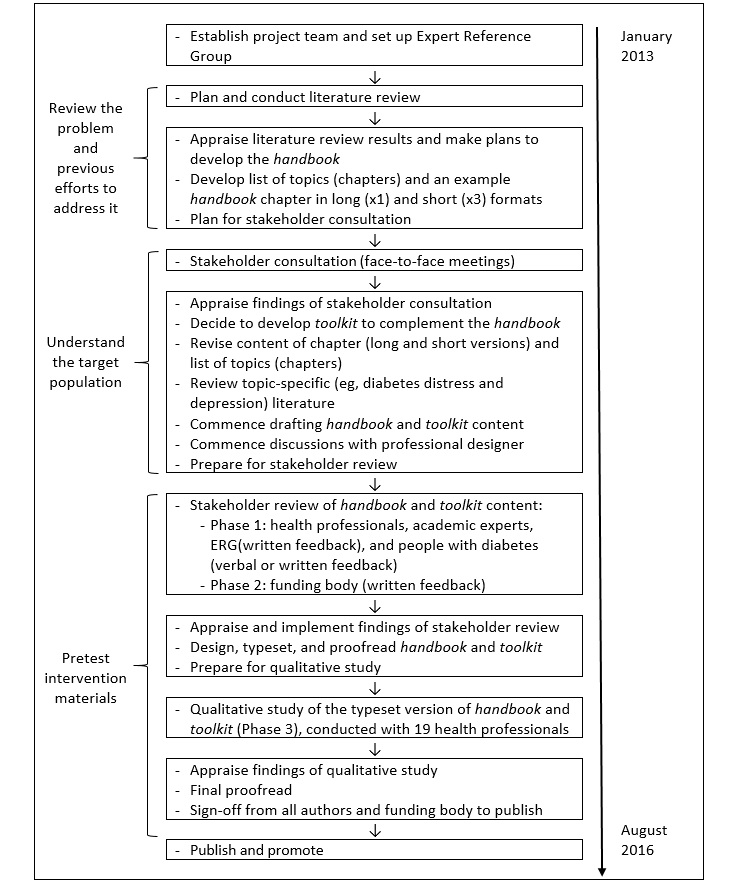
Summary of the formative evaluation of the Diabetes and Emotional Health handbook and toolkit.

To develop the *handbook* and *toolkit*, we used a formative evaluation approach, that is, “a set of activities designed to develop, identify and test program materials and methods … [which] occurs as a part of program planning and occurs before any elements of the program are implemented” [24]. We selected this approach to enable us to shape the resources in accordance with the literature, best practice clinical recommendations, and stakeholder consultations, to meet the needs of diabetes health professionals. The formative evaluation steps we applied (ie, review the problem, understand the target population, and pretest the intervention material) [24] are shown in Figure 1 and described in the following sections. The methods of each phase were informed by the results of the previous phase (ie, an iterative process). The decisions made after each phase are described in each corresponding results section.

### Establish Project Team and Set Up Expert Reference Group

To begin, we established a project team that included health and clinical psychologists and researchers with expertise in the psychological aspects of diabetes. We also established a multidisciplinary Expert Reference Group (ERG), representing various stakeholders (eg, general practitioner, endocrinologist, diabetes nurse educator, psychologist, key organizations, and people with diabetes). The project team worked collaboratively throughout the project, meeting regularly, holding workshops to discuss and progress ideas, and constructively reviewing each other’s work. We engaged the ERG at least quarterly to discuss plans and progress.

### Review the Problem and Previous Efforts to Address It: Literature Review

In 2013, we conducted a narrative literature review to investigate 7 research questions (see Multimedia Appendix 1). We developed these research questions using a stepwise approach, to help formulate an evidence base for developing a resource to support diabetes health professionals with providing psychological support. We engaged our ERG in the process of formulating the research questions. We searched MEDLINE, Scopus, Google (for gray literature), and the reference lists of relevant literature. Peer-reviewed narrative and systematic reviews and meta-analyses, empirical research (quantitative and qualitative), expert commentaries, reports, and clinical guidelines were consulted. The search was limited to literature relevant to adults with type 1 or type 2 diabetes, published in English from the year 2000 onward. Search terms varied according to the research question (eg, Diabetes and Psychological). Truncations and synonyms were used as appropriate.

The literature review confirmed the need for practical, user-friendly, and evidence-based resources; thus, we decided to develop a handbook to meet this need (See “Review the Problem and Previous Efforts to Address It: Literature Review” in the Results for further information).

### Understand the Target Population: Stakeholder Consultation

From late 2013 to early 2014, JH and LB consulted with each ERG member individually to (1) gain a deeper understanding of their current practice related to diabetes-related psychological care and (2) ascertain their needs (individual and professional, content and design) for the *handbook*. The consultations were unstructured to ensure the relevance to all ERG members and used funneling (moving from broad to specific topics). Before the consultations, the project team agreed on a general list of topics to discuss, but they also agreed to allow the stakeholders to expand on any relevant topic (consistent with an unstructured consultation approach). For example, after the person had spoken, unprompted, about their experiences and needs, they were shown pre prepared examples of draft *handbook* content, in long (*chapter*) and short (3 *summary versions*—5 A’s model, flowchart, and factsheet) formats. We explored their preferred version, positive and negative aspects of each version, how each version would meet their needs (or not), and how we could adapt each version to better suit their needs. We also built on topics raised by other ERG members (eg, “Someone else suggested X, what do you think?”) and presented a draft list of topics (chapters) to check relevance and completeness. We made comprehensive notes during the consultations. JH examined all notes for commonalities and differences in views, then summarized the results (see “Understand the Target Population: Stakeholder Consultation” in the Results) and discussed them with the team. We reported back to the ERG to validate the findings and seek agreement for proposed plans and decisions, which included a decision to develop both a *handbook* and a *toolkit*, to meet the varying needs of different health professional disciplines (see “Understand the Target Population: Stakeholder Consultation” in Results).

### Pretest Intervention Methods and Materials: Phases 1–3

#### Phase 1: Review by Professionals and People With Diabetes

A review of each completed draft chapter and its corresponding summary and questionnaire cards was conducted to ensure that the content was consistent with best practice and recent evidence and met the needs of the intended audience. The factsheet (part of the *toolkit*) development is outside the scope of this study, but stakeholders also reviewed these resources. In total, four professionals reviewed each chapter (plus the corresponding summary and questionnaire cards): one health professional, one academic expert, and two ERG members. We invited the reviewers on the basis of their clinical or academic experience and relevant publication record. The reviewers were multidisciplinary, including psychologists, endocrinologists, general practitioners, dietitians, and credentialed diabetes educators. An adult with type 1 or type 2 diabetes also reviewed each chapter, to ensure they deemed the content as appropriate (eg, language use, references to people with diabetes, their experiences, and suggested strategies or techniques).

Owing to the significant time and work required to review the chapters (estimated minimum 3 hours), we offered remuneration to all reviewers. We provided the reviewers with background information (eg, aim, scope, content, and target population), instructions (eg, scope for the review), and a nontypeset copy of the chapter and summary (both annotated with guiding questions). JH prepared and provided guiding questions relevant to each reviewer’s background to ensure the reviews were within the scope of the individual’s expertise (eg, medical content was not the responsibility of mental health professionals or people with diabetes). The professionals provided written feedback, while the people with diabetes provided written or verbal feedback. JH met (face-to-face or telephone) with those who opted to provide verbal feedback, making comprehensive notes during and immediately after the conversation. Once all reviewers provided feedback, we consolidated the feedback for each chapter into a single document for team review and implementation.

#### Phase 2: Review by Funding Body

The National Diabetes Services Scheme (NDSS; funding body) required its Medical, Education, and Scientific Advisory Council (MESAC) to review and approve the resources before publication. We submitted a standardized form with background information in addition to the nontypeset *handbook* and *toolkit* drafts. The MESAC reviewed the draft *handbook* and *toolkit* (summary and questionnaire cards) to provide tracked changes and comments. The factsheets were also subject to MESAC review, but further details of their development and review is beyond the scope of this paper.

#### Phase 3: Interviews With Health Professionals

As a final step, we undertook a qualitative study of the *typeset versions* of the *handbook* and *toolkit* (summary and questionnaire cards). We aimed to (1) collect feedback about the *handbook’s* content, structure, and usability and (2) understand health professionals’ perspectives on implementing the strategies described in the *handbook*. We developed a Web-based screening survey and a semistructured telephone interview schedule. The survey included demographic and clinical characteristics and brief questions, such as confidence to talk about and assist with the emotional aspects of diabetes (measured on a Likert scale from 1=not at all confident to 5=very confident). The interview schedule included questions about the resources (eg, overall impressions of *handbook*, what they learned, and implementation plans).

We promoted the study via newsletter advertisements and direct emails to health professionals who had previously registered their interest. Participants were eligible if they worked in Australia as a general practitioner, endocrinologist, credentialed diabetes educator, nurse, or dietitian and consulted with at least 10 adults with diabetes weekly. About 2 weeks before the interview, we sent consenting participants a typeset copy of the *handbook* and summary cards, details of the study procedure, and a summary interview guide. They were asked to read the “Introduction;” “How to use this *handbook* and *toolkit;”* and their choice of at least one of the chapters focused on a particular psychological problem (ie, Chapters 3 to 8) and the associated summary card(s).

A researcher who did not develop the *Diabetes and Emotional Health* resources (AB) conducted the interviews. The interviews were audio-recorded and transcribed professionally. Subsequently, AB summarized responses and categorized quotes from the transcripts according to the interview questions. CH checked the categorization and summaries, then prepared a report of the findings, which AB, JH, JS, and the ERG reviewed and approved.

## Results

### Review the Problem and Previous Efforts to Address It: Literature Review

The literature review demonstrated that psychological problems are common among Australian adults with type 1 and type 2 diabetes [25-27]. Furthermore, Australian adults with diabetes attending tertiary diabetes clinics respond well to the use of psychological screening questionnaires [27,28]. International guidelines recommend attention to and routine screening for psychological problems as a part of routine care [29] yet health professionals report lacking resources, training, and confidence to do so [19,30]. We presented these results in a report that the ERG reviewed and approved. These findings and “Lessons Learned and Actions” are summarized in Multimedia Appendix 1.

On the basis of the literature review findings (see Multimedia Appendix 1), we decided to develop a practical, user-friendly, and evidence-based resource (ie, the *Diabetes and Emotional Health handbook*). The *handbook* would cover a range of diabetes-specific and general psychological problems common to adults with type 1 or type 2 diabetes, which affect their diabetes self-care and outcomes and quality of life. It would provide health professionals with practical tools and strategies for identifying, communicating about, and addressing psychological problems. The ERG supported our proposed plans. They also pointed out that health professionals often have limited time and would appreciate short (eg, 1 page) summaries, with an option to access more detail and background information in the *handbook*.

**Figure 2:**
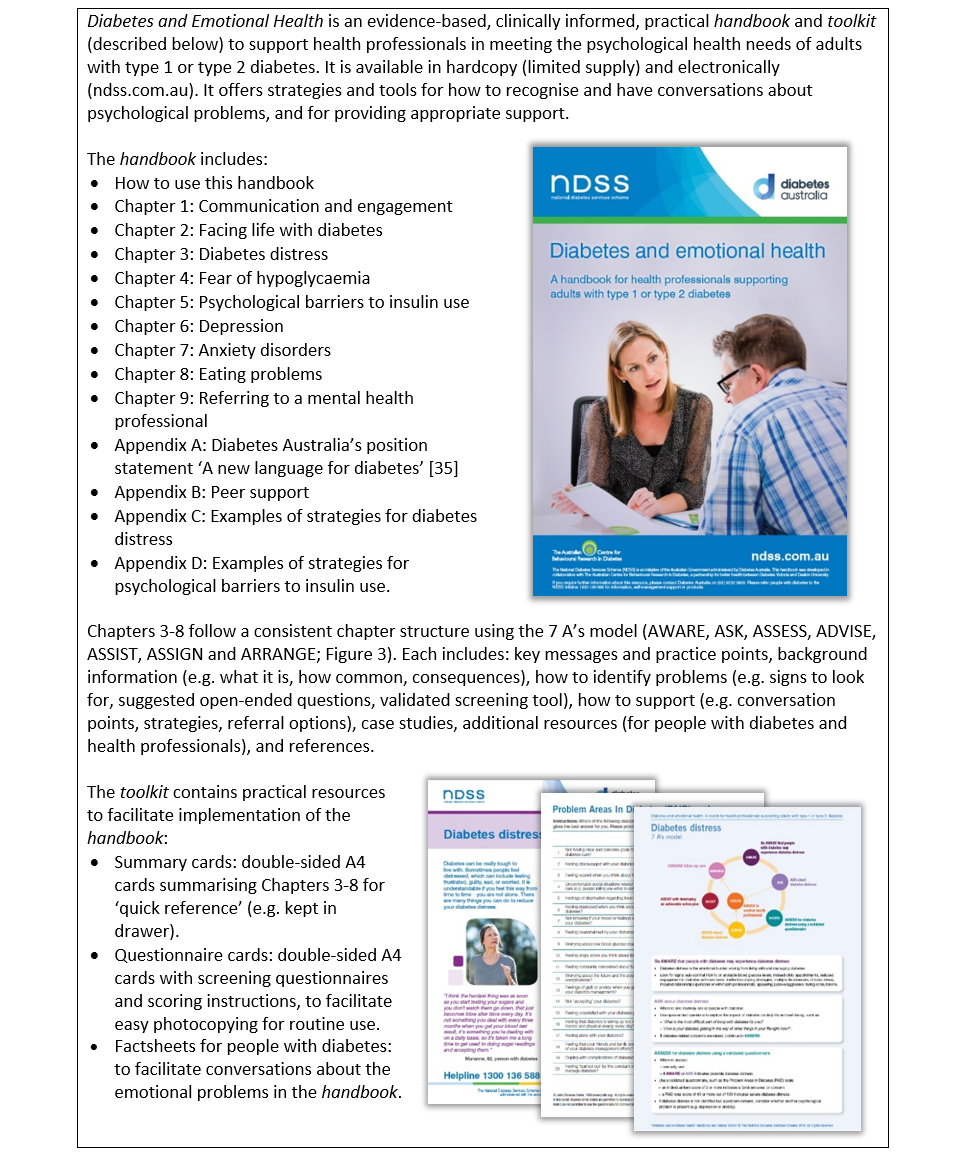
Overview of the Diabetes and Emotional Health handbook and toolkit.

**Figure 3:**
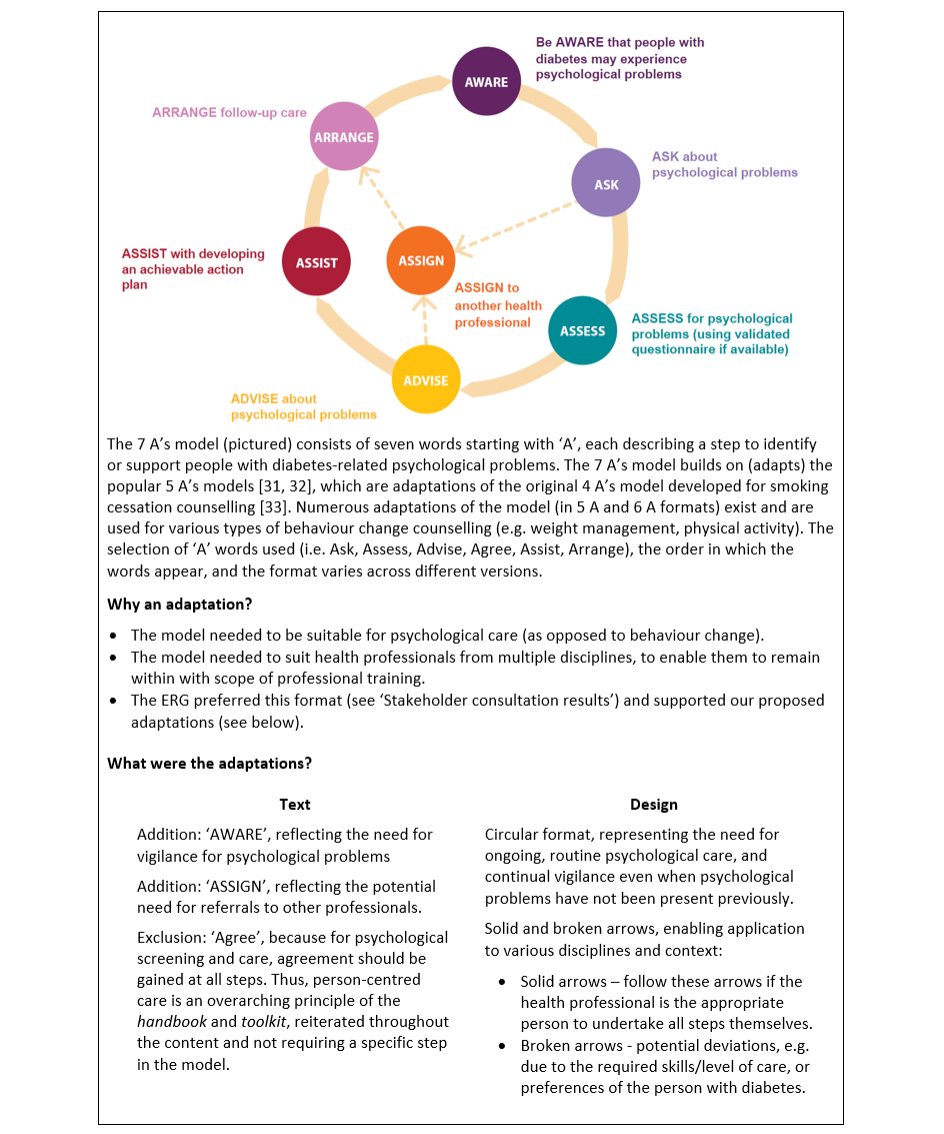
Overview of the 7 A’s model.

The literature review informed the *handbook* planning and content. We began by brainstorming a list of proposed topics (ie, chapters; see Figure 2) and chapter content (proposed headings and sections). Drawing on content-specific literature, we prepared a draft chapter, proposing a standardized format with the following headings (structure): key messages, prevalence, risk factors, impacts, what to look for, treatment or management, case studies, resources, and a copy of a validated screening questionnaire. We also investigated formats for the summaries and prepared drafts in three formats (5 A’s model, flowchart, and factsheet). We selected these potential formats because they are common layouts in other resources (eg, the 5 A’s model appeared in existing Australian health professional guidelines) [31]. The 5 A’s model is a well-cited adaptation of the 4 A’s model (see Figure 3) [32-34].

### Understand the Target Population: Stakeholder Consultation

The ERG consultations facilitated greater understanding of the roles of different health professional disciplines and their needs. A summary of the key results is included in Table 1. The consultations highlighted the diverse needs (eg, learning styles and scope of practice) and preferences of the ERG members. While some preferred the detail of the chapter, others preferred the brevity of the summaries. The 5 A’s model was the preferred summary format. The ERG suggested to develop complementary factsheets for people with diabetes about the emotional problems in the *handbook*.

**Table 1:** Results and actions arising from stakeholder (Expert Reference Group) consultations

Topic	Results	Actions
Assessment of psychological health in clinical practice settings	Each practicing health professional had a different preferred style, for example: Enquiring through conversation and open-ended questions, because they believe a structured questionnaire might divert the focus of the conversation from the agenda of the person with diabetes to the agenda of the health professional. Routine screening using short questionnaires, because they believed it is easy to miss problem areas in conversation, eg, the person with diabetes might not raise it themselves. They believed that asking people to complete the screening questionnaires in the waiting room (before the consultation) is appropriate. They agreed that introduction of the questionnaire to the person with diabetes is important (eg, to explain the purpose and that completion is optional). Annual assessment (eg, for diabetes distress using the PAID^a^ scale), because it forms part of an annual holistic approach to care and is acceptable to people with diabetes.	As there is no evidence to support one approach over another, the *handbook* caters to these different styles. It provides examples of different ways to incorporate psychological screening into routine clinical practice.
Feedback about the *long version* (sample chapter) and *summary versions* (ie, sample 5 A’s model, flowchart, and factsheet)	*Long version (sample chapter)*: Some thought the amount of detail (length) in the example chapter was appropriate and that there was a good balance of bullet points and sentences. They believed that some health professionals would want this level of detail, but that others may not have time to read it. So, it would be important to find a balance between detailed information and 1-page summaries. They offered various suggestions for presentation (eg, develop electronic and printed versions, use a PDF rather than CD for the electronic version). *Summary versions*: Of the 3 short formats provided, the 5 A’s model was preferred because it was a simple, step-by-step guide and would be familiar to many health professionals. They made minor suggestions for improvement (eg, reducing text and design elements). Summary cards would be useful to put on wall or in top drawer (for quick accesses). They suggested and agreed that factsheets for people with diabetes would be useful (to facilitate conversations to distribute in consultations and waiting rooms).	The *handboo*k contains detailed information, while the *toolkit* contains double-sided summaries, copies of validated questionnaires, and factsheets for adults with diabetes. It caters to all expressed needs and preferences. The 5 A’s model (later adapted to a 7 A’s model, see Figure 3) is a key element of the *handbook* and summary cards (consistent structure). Electronic (PDF) and hardcopy versions of the *handbook* and *toolkit* are available (from ndss.com.au).
Topic-specific feedback	They offered suggestions regarding topics to include and exclude and questionnaires to include and exclude.	We considered and included the suggestions as appropriate (eg, within scope of project and supporting evidence).
Language considerations	Use plain language in communications with and for people with diabetes. Define commonly used words and use terms consistently. Avoid referring to “patients.”	We made efforts to ensure appropriate and consistent language use, and in accordance with published recommendations [35]. The *handbook* includes a Glossary of terms. The *handbook* and *toolkit* were professionally copyedited and proofread. For the factsheets, this included plain language editing.
Ideas for future stakeholder consultation	Include people with diabetes in the next phase of consultation. A suggestion was offered for implementing stakeholder consultation (based on a process which worked well for another organization when developing factsheets)—to prepare unformatted drafts and email it to stakeholders (eg, professional bodies and consumers), for written responses or tracked changes. Give a few guiding questions in the cover letter then leave it “open”.	We adapted (to suit the project) and implemented the suggested consultation method. Health professionals, academic experts, and people with diabetes reviewed the *handbook* and *toolkit* (see “Phase 1: Review by Professionals and People with Diabetes” in the Methods and Results).
Suggestions for dissemination and future work	Consider attaining endorsement of the *handbook* by relevant professional bodies. Consider developing training to complement the *handbook* and offer professional development points.	We did not pursue endorsements, owing to complexity of the process and because the *handbook* and *toolkit* would adopt the strong brand and endorsement of the funding body. We informed relevant professional bodies of the resources (enabling promotion to their members). We pursued funding opportunities to enable development of Web-based health professional training.

^a^PAID: Problem Areas In Diabetes.

On the basis of the consultation results, we decided to develop both a *handbook* and *toolkit* to meet the diverse needs of the various health professionals involved in routine diabetes care. The *handbook* would provide detailed information, with each chapter using a consistent format to enable quick reference (eg, consistent headings and structure, guided by the 5 A’s model, which was the preferred *summary* format) [31]. The *toolkit* would provide practical resources for use in clinical consultations, that is, 1-page summary cards of each chapter (focused on the 5 A’s model), copies of validated questionnaires, and factsheets for people with diabetes.

Drafting the *handbook* and *toolkit* was a collaborative process. We divided the topics among the individual authors (CH, JH, LB, and JS) to lead preparation. For some chapters, the writing was shared (eg, by multiple authors or with a contributor from outside the core authorship team). Overall, a minimum of 3 authors contributed to each draft chapter. We drew upon clinical practice guidelines, published peer-reviewed literature, the findings of the ERG consultation, our own clinical and research experience, and iterative ongoing discussions (with the ERG and team). We held writing workshops regularly to discuss writing progress and content and to make adjustments for consistency between chapters.

### Pretest Intervention Methods and Materials: Phases 1–3

#### Phase 1: Review by Professionals and People With Diabetes

The reviewers (n=37) provided positive and constructive feedback. Many suggestions for improvement were minor and most was chapter specific (eg, add a reference, adapt a suggested strategy, or change a word). Several reviewer suggestions were outside the project scope (eg, include information about complex psychiatric conditions or for carers of people with diabetes) or included elsewhere in the *handbook* (eg, information about cultural and linguistic diversity). Some suggestions conflicted with those of other reviewers; this diversity reflected the multidisciplinary nature of the content and review, and individuals’ views and areas of interest. A common criticism was the long chapter length, but there was no consensus among the reviewers about what information to remove; they considered all content important and had differing opinions about the most valuable content.

We held several whole-day team workshops to compare, discuss, and review the feedback. This included discussion of conflicting feedback and views. To overcome conflict, we discussed and collaboratively made pragmatic decisions (eg, could we add a text box or bullet point, or refer the reader to another page or chapter?). We sought clarification from reviewers as needed (eg, we wanted clarification or further information). Significant revisions were required for 2 chapters: “Communication” and “Facing life with diabetes.” Finally, the author who drafted the chapter implemented the agreed revisions.

Where relevant, we implemented feedback across multiple chapters of the *handbook and toolkit*. Examples include the following:

- Structured case studies—we had developed 2 versions of the first 2 chapters reviewed (“Psychological barriers to insulin use” and “Depression”), one “structured” in accordance with the 7 A’s model and one “unstructured.” The reviewers indicated that the structured version was preferable, so we structured all other case studies in this manner.
- Editing—we developed a style guide to ensure consistency across the handbook in reducing unnecessary wordiness, improving readability or understanding, and improving the language (to be more positive, empowering, inclusive, supportive, and consistent).
- Quotes from people with diabetes—we added these to demonstrate examples of concepts in the handbook.

#### Phase 2: Review by Funding Body

The MESAC viewed the *handbook and toolkit* favorably. The feedback was mostly minor and chapter specific (eg, rephrasing a sentence or changing a word), reflecting the rigorous development and review process. MESAC also commented on the *handbook* chapter length but could not make specific suggestions for shortening them.

We implemented the requested changes as appropriate (eg, rewriting some sections to reduce wordiness), resulting in a *final version* (described in Figure 2). Some comments were outside the scope of the project and could not be included in the *handbook* and *toolkit*. For example, suggestions to include information for “carers” (ie, family and friends of people with diabetes) were not implemented, as it would have required different information and consultations. At least three authors (CH, JH, and JS) reviewed this final version. We documented the changes made and provided the MESAC with a written response and copy of the final version. The MESAC approved the final version.

#### Phase 3: Interviews With Health Professionals

We interviewed 19 participants, of 25 health professionals who volunteered. The participants included 9 nurses and 6 dietitians (of whom 7 and 1 were credentialed diabetes educators, respectively), 2 general practitioners, and 2 endocrinologists. The participants worked in urban or metropolitan (9/19, 47%) and regional or rural (10/19, 53%) settings. Most (16/19, 84%) worked in a multidisciplinary health service that did not include a mental health professional in the team. They reported varying levels of confidence to talk about (median 3, range 2-5) and assist with (median 3, range 2-5) with diabetes-related emotional problems; 37% (7/19) had used a questionnaire in clinical care to assess emotional health. The 6 who did not participate could not be reached at the time of interview. They had similar confidence to talk about and assist with emotional problems, compared with the participants, but none had used questionnaires.

The chapters selected by participants were fear of hypoglycemia (n=7), diabetes distress (n=5), eating problems (n=4), psychological barriers to insulin use (n=1), depression (n=1), and anxiety disorders (n=1). Most also read other chapters of the *handbook*.

Participant quotes are included in Table 2. Overall, the *handbook* and *toolkit* were viewed favorably as well-written, easy to read and understand, and easy to navigate, with consistent chapter structure and good design elements (eg, use of colors, fonts, and boxes). Perceived *highlights* were practical elements, such as the examples of open-ended questions, validated questionnaires, case studies, 7 A’s model, and summary cards. For example, 1 nurse practitioner described how she valued the summary cards in the *toolkit*, “Loved the summary cards and felt that this was the handbook's biggest strength.” Participants gave very few suggestions for improvement; the most common criticism was the chapter length, but they were unable to offer suggestions to shorten it and they appreciated its comprehensiveness.

The participants reported how the *handbook* raised their awareness about the role of health professionals in attending to the psychological aspects of diabetes, which encouraged *self-reflection* on their practice. This is demonstrated well by a Nurse Practitioner-Credentialed Diabetes Educator who said, *“*I think it's a really good way to actually reflect back on...your own thoughts and about how you actually engage in asking about diabetes distress.” The *handbook* also helped to build *self-confidence*, affirming for some that their current clinical practice was *on track*. For example, a dietitian found validation in the *handbook’s* message that health professionals are often best placed to provide support for diabetes distress*,* “And, the other comment that’s interesting is that diabetes distress is best managed within the context of diabetes care...[it] improves my confidence and I think ‘yeah – that’s what I do with people.’”

The participants commented how the *handbook* could *influence clinical practice*—some had already implemented aspects of the *handbook* (eg, asking more frequently about how the person with diabetes feels), whereas others planned to make changes (eg, implementing routine assessment for diabetes distress and trying new strategies for observed problems). For instance, 1 Credentialed Diabetes Educator-Registered Nurse gave an example of how she asked about a person’s well-being after reading the *handbook* and subsequently provided a listening ear and psychology referral, “I was reading the handbook before the patient came in two days ago and I said to her, 'How are you going? How are you feeling about everything? Are you managing things?’...I realized it was a real can of worms...I did refer her on...”

The participants identified some possible *barriers to implementation*, including a lack of referral options (to mental health professionals, particularly with diabetes expertise) and costs of intensified follow-up (eg, calls or text messages, when needed). For example, an endocrinologist commented “I think the problem with the psychology referral is that...people with a specific interest in diabetes are few and far between.” Notably, the participants felt empowered not only to implement the resources but also to *spread the word*, by recommending it to other health professionals or even training others to use it. For example, one dietitian explained, “I’ve talked to my boss and I wouldn’t mind doing a bit of an information session to other dietitians and coaches about it.” They also offered suggestions for future work (eg, *promotion* and *training*), such as this from a Credentialed Diabetes Educator-Registered Nurse, “I think it needs to go into the Graduate Certificate for Diabetes Education. I think it needs to go to medical students and...pharmacy students as well...” Some felt that the *handbook* was sufficient as a stand-alone resource to supplement participants’ existing professional skills and experience. Others considered that training would be useful to help build confidence or to enhance specific skills (eg, introducing, scoring, and responding about questionnaires).

Given the positive feedback and lack of major concerns, CH, JH, and JS finalized the content and typesetting. Electronic and hardcopy versions of the *Diabetes and Emotional Health* handbook and toolkit were published in print and on the Web [23]. Promotion of the resources commenced in August 2016, at an Australian diabetes conference, and is ongoing.

**Table 2:** Qualitative study evaluation of the handbook and toolkit by health professionals.

Topic	Example quotes
Perceived highlights	“Most useful section was the ASK section as it gave practical tips on asking questions that will bring up any issues.” (Dietitian, male, eating problems) “I really liked the quotes from practice nurses and quotes from health professionals and quotes from consumers. I thought that was a nice value add. And, I liked the ‘ABCs of effective communication.’” (Dietitian, female, diabetes distress) “Loved the summary cards and felt that this was the *handbook*'s biggest strength.” (Nurse practitioner, female, depression) “Irena [case study]…that would be a very common scenario…I thought it was a great example, and it's brief, it's not too lengthy. And then it sets out the 7 A’s framework in each section of what you can do…. so it's easy for the practitioner to work with that.” (Registered nurse-diabetes educator, female, fear of hypoglycemia) “I’m quite visual in my learning, so, when I’m trying to think about structures in my head, I think visually and I like colors… I like the approach of it.” (Registered nurse, female, diabetes distress) “The structure is well laid out and it certainly follows the 7 A’s model very well. All the chapters have done that, which is exactly what you want. You want consistency so that the reading is streamlined and everyone who reads it can access what they want very quickly.” (GP, male, fear of hypoglycemia)
Role of health professionals	“I think that the one thing that the *handbook* says… that comes out in every chapter, at every appointment, ask about their well-being. Don't just assume they're okay …ask the question.” (Credentialed diabetes educator-registered nurse, female, fear of hypoglycemia) “For me, this book has really normalized that our role is working with a whole person, their emotional health and their physical health… sometimes in the rush of everything, and especially in a tertiary setting, you can get quite focused on the physical aspect of somebody who’s acutely unwell… I found it a really useful tool to just ensure that we all keep in mind that we’re working with a person.” (Registered nurse, female, diabetes distress) “I think the point that kept coming through… most people actually do want to go to a member of their diabetes team to talk about this. So, if my question was, you know, is this my role? Well, yeah, it is. They’re seeing it as my role.” (Dietitian, female, diabetes distress)
Encourage self-reflection	“I think it's a really good way to actually reflect back on… your own thoughts and about how you actually engage in asking about diabetes distress.” (Nurse practitioner-credentialed diabetes educator, female, diabetes distress) “I used the Diabetes Distress chapter more for like, a concrete reflection on my practice.” (Registered nurse, female, diabetes distress) “I discussed it with a Type 1 client and we've made an arrangement that next time that she has an appointment that we will discuss her views on this, because it's something that I feel that I could do better on it as a clinician.” (Credentialed diabetes educator-registered nurse, female, fear of hypoglycemia)
Build self-confidence	“And, the other comment that’s interesting is that diabetes distress is best managed within the context of diabetes care… [it] improves my confidence and I think ‘yeah – that’s what I do with people’ and just talking through their feelings and what’s an issue for them – that I’m doing the right thing… Which is such a feeling of relief really.” (Dietitian, female, diabetes distress) “That 7A’s thing, it really worked. I was nicely surprised. Yeah. And, it felt like I didn’t do anything different to what I normally do, but, I had a language for it. So, that I could work it through for myself.” (Registered nurse, female, diabetes distress)
Influence clinical practice	“I was reading the *handbook* before the patient came in two days ago and I said to her, 'How are you going? How are you feeling about everything? Are you managing things?...I realized it was a real can of worms … it was not just about her diabetes. It was about feeling overwhelmed…She actually ended up saying to me, ‘The only reason why this conversation's coming out is because you asked me how I am. Had you not asked me, I wouldn't tell you that I'm getting depressed… I am noticing that I am mood swinging and I don't know whether it's my stage of life, and problems which are hormonal, or whether it's depression’...In that situation I did refer her on because I feel that she's been missed...” (Credentialed diabetes educator-registered nurse, female, fear of hypoglycemia) “The 7A’s tool itself, in working with this client – it let me look at structuring how to work both with … the physical aspects and the emotional responses together. It was really useful.” (Registered nurse, female, diabetes distress) “I really think that part of my practice I need to do more of regularly is start to use the PAID scale and particularly for everyone, not just picking some people but just doing it on everyone, not all the time, but doing it. But, I really think it will actually bring out a lot of things that both sides of the party didn’t realize or think about.” (Nurse practitioner-credentialed diabetes educator, female, diabetes distress) “I can think of a number of people I see where I've recognized fear of hypoglycemia and there's information within that chapter which may help me work through that fear with them.” (Endocrinologist, male, fear of hypoglycemia)
Spread the word	“I actually showed one of my neighbors who is a pharmacist and I was discussing with him, on chapter 5, how good I thought it was to do decisional balancing with someone who is reluctant to start insulin.” (Credentialed diabetes educator-registered nurse, female, fear of hypoglycemia) “I’ll be actively promoting it as a resource and I’ll be wanting our GPs to have it available to them and I’ll be promoting it heavily.” (Credentialed diabetes educator-registered nurse, female, eating problems) “I’ve talked to my boss and I wouldn’t mind doing a bit of an information session to other dietitians and coaches about it… summarize the importance of dealing with the emotional aspect of diabetes and then point to some of these summary cards and… the *handbook*.” (Dietitian, female, diabetes distress)
Barriers to implementation	“I think the problem with the psychology referral is that… people with a specific interest in diabetes are few and far between… But, it would be really nice to know which psychologists for instance, in our area, are interested in dealing with patients with diabetes and the same with psychiatrists. I think that’s where I struggle clinically.” (Endocrinologist, male, fear of hypoglycemia) “You know, unless you are lucky enough to have a psychologist that works and is employed within a practice and they don't charge a gap, people can’t afford that.” (Nurse practitioner-credentialed diabetes educator, female, diabetes distress) “Regarding diabetes distress, it's [*handbook*] encouraging us as health professionals to be able to get on the telephone to follow up, or to arrange all this follow up, but that's not remunerated under Medicare… Under the Medicare system and under the care planning arrangements that we have, I might have one visit allocated, so it doesn't give me necessarily a follow-up visit without the patient then being out of pocket...” (Credentialed diabetes educator-registered nurse, female, fear of hypoglycemia) “We have 20 minute appointments and have to deal with the medical side of diabetes and then to deal with psychological side too, we probably can designate maybe five minutes – ten minutes, if we’re lucky… we need the numbers and the resources and things at our fingertips… I think it’s fantastic and it definitely is needed but we need to have that information right there or in a website form where we can just go click.” (Endocrinologist, female, fear of hypoglycemia)
Suggestions for promotion	“Getting it out there, is the key… a good advertising campaign, and that's probably best orchestrated through presentation of some of the work in the book at the various diabetes-related clinical meetings that occur around the countryside...” (Endocrinologist, female, fear of hypoglycemia) “I think it needs to go into the Graduate Certificate for Diabetes Education. I think it needs to go to medical students and…pharmacy students as well…” (Credentialed diabetes educator-registered nurse, female, fear of hypoglycemia)
Training needs and ideas	“… actually getting people to physically do the questionnaire themselves or give them a case study scenario so that they’re doing it from the perspective of their person… it’s really important to be familiar with the tool. But, also scoring. So, it’s one thing to give people a questionnaire to fill out but to then be able to score it on the spot and give them some feedback.” (Dietitian, female, diabetes distress) “…as part of the training something along the lines of a mentoring or relationship...you might, as part of the training process, bring along how you do things, case studies, that part of it is meeting and talking with a peer.” (Nurse practitioner-credentialed diabetes educator, female, diabetes distress) “...I think they [chapters] could all be incorporated into one workshop, covering how to ask questions and what to do with the information once you've got it and how to build trust, all that sort of thing.” (Nurse-diabetes educator, female, eating problems) “…practical case studies based. The other thing that works well for medical practitioners is webinars and well, something where people can be at home listening to or do it in their own time or, perhaps type questions in.” (Endocrinologist, female, fear of hypoglycemia) “We need to make the assessment of emotional health a compulsory part of guidelines… because they all tick off these other things on their list. We have to test their cholesterol levels twice a year. We're going to do their HbA_1c_ twice a year… but no one actually asks them about how they are.” (Dietitian, female, eating problems)

## Discussion

### Principal Findings

While guidelines recommending psychological care in diabetes have existed for 25 years [5], to our knowledge, the *Diabetes and Emotional Health handbook* and *toolkit* [23] represents the first attempt to develop evidence-based, clinically informed, freely available, practical resources for multidisciplinary diabetes health professionals supporting the emotional health of adults with type 1 and type 2 diabetes. These resources are a tailored response to the expressed unmet needs of health professionals, who cite lack of resources and confidence to address diabetes-related emotional problems as significant barriers to providing holistic diabetes care. The *handbook* offers strategies and tools for recognizing psychological problems and providing support for them. The *toolkit* contains practical resources to facilitate implementation: chapter summary cards, questionnaires, and factsheets for people with diabetes. The *handbook* and summary cards implement the 7 A’s model. A model such as this provides a memorable acronym for application in busy health settings and is consistent with the expectations of people with diabetes about support from health professionals [34]. The 7 A’s model is a useful framework to provide a consistent and logical structure with a clear path to implementation in clinical practice. The reviewers and qualitative study participants favorably viewed its application in the *handbook*.

Formative evaluation is an essential first step for developing high quality and effective interventions that are acceptable to the target population [24]. In this case, formative evaluation helped us to comprehensively explore the problem, while ensuring accountability and quality control. The formative evaluation approach is a key strength of the resources*.* The inclusion of several stages of end-user (health professionals) and stakeholder (eg, academic experts and people with diabetes) consultation means that we are confident that the resources align with expressed needs and published evidence. Given our combined expertise, we could have developed these resources with less consultation, which would have been less resource and time intensive. However, we would have been less confident with the final product and the rigorous review process was well received by and inspired confidence among stakeholders and potential users [24]. The methods described in this study demonstrate how formative evaluation can inform the development of high quality, evidence-based resources, and the processes described herein may be valuable for informing the development of similar resources in other areas.

These resources are important stepping stones toward more consistent implementation of clinical practice guidelines and better integration of psychological health into routine diabetes consultations. As described by 1 qualitative study participant, the *handbook* has “really normalized that our role is working with a whole person, their emotional health and their physical health.” When we commenced this project, there was no specific Australian guideline for the psychological care of people with diabetes. In addition to developing *Diabetes and Emotional Health*, we concurrently advocated for recognition of psychological care within existing Australian guidelines to align them more closely with international recommendations. Since then, recommendations for routine screening for depression and diabetes distress have been included in the Australian General Practice Management of Type 2 Diabetes guidelines [7,9]; the Australian guidelines for type 1 diabetes [36] are yet to be revised. Importantly, mental health has been recognized in the Australian Government’s National Diabetes Strategy: 2016-2020 [37]. Moving forward, we continue promoting these resources via seminars, workshops, conferences, and social media. These resources are part of the training curriculum for the next generation of credentialed diabetes educators (eg, at Flinders University and Deakin University). We have developed, and are currently evaluating, a Diabetes Distress e-Training for health professionals, on the basis of the *handbook* and *toolkit* content. Additionally, we have collaborated internationally on a Diabetes UK adaptation of the *handbook* and *toolkit* to suit the UK health care context*,* which is now available on the Web [38]. We have also been able to address the resource gap raised by the MESAC (see “Phase 2*:* Review by Funding Body*”* in the Results) by subsequently developing a factsheet for family and friends of people with diabetes [39].

We acknowledge that there is more work to do. For example, a limitation of this study was the exclusive focus on adults with type 1 and type 2 diabetes. We selected these 2 groups as they represent the largest populations in need of psychological support, and our previous research had focused largely on adults [25-28]. However, there is a need for similar resources to enhance support for children and adolescents with diabetes (and their families) and women with gestational diabetes. Furthermore, the diabetes-related psychological needs of specific subgroups (such as Aboriginal and Torres Strait Islanders, culturally and linguistically diverse communities, people with psychiatric conditions, and people with disabilities) are under-researched and important areas for future work. Evaluation of the process and impact of the real-world implementation of the resources would also be valuable in the future [24] but was not within our project scope.

### Conclusions

Diabetes and Emotional Health is a practical, evidence-based, clinically informed handbook and toolkit developed in consultation with end-users and other stakeholders. The findings of our formative evaluation suggest that the resources are comprehensive yet user friendly, addressing the previously unmet needs of multidisciplinary health professionals, enabling professional development and supporting real-world implementation of clinical practice guidelines related to the psychological care of people with diabetes.

### Practice Implications

Diabetes and Emotional Health provides health professionals with practical information and tools required to implement clinical practice guidelines related to the psychological aspects of diabetes. More than 1000 hardcopies have been distributed to Australian health professionals and more than 1400 electronic copies have been downloaded. The resources remain freely available on the Web. [23] Health professionals find the handbook and toolkit useful for their clinical practice, are implementing them, and are taking ownership of them (eg, discussing with others and making plans to train others). These resources are likely to have clinical utility internationally (until international adaptations are developed), owing to the evidence-based content, robust stakeholder review, and shared goals with international clinical practice guidelines (holistic and person-centered care and attention to psychological problems). Similarly, our adapted 7 A’s model may have clinical utility for routine screening and monitoring for other problems (psychological or other) as part of a person-centered approach to routine care.

## Appendix

Multimedia Appendix 1 Literature review questions, results, lessons, and actions.

## Abbreviations

ERG: Expert Reference Group
MESAC: Medical, Education, and Scientific Advisory Council
NDSS: National Diabetes Services Scheme
