# Supplementary material for: The Diabetes and Emotional Health Handbook and Toolkit for Health Professionals Supporting Adults With Type 1 and Type 2 Diabetes: Formative Evaluation
Source: JMIR Form Res. 2020 Feb 21;4(2):e15007. doi: 10.2196/15007 (PMC7060499; doi:10.2196/15007)
Supplement: Multimedia Appendix 1 [file formative_v4i2e15007_app1.docx]

Multimedia Appendix 1. Literature review questions, results, lessons, and actions.

| Research Question | Results^a^ | Lessons learned and Actions |
| --- | --- | --- |
| 1. What is the prevalence of psychological problems in adults with type 1 and 2 diabetes, and what are its implications (eg, for diabetes management or outcomes and well-being)? | - Diabetes-specific and general psychological problems (eg, depression and diabetes distress) are common among adults with diabetes [1, 2]. For instance, 1 in 5 experience severe diabetes distress and 1 in 4 experience moderate-to-severe depressive symptoms [3]. Other common problems include fear of hypoglycemia, psychological barriers to insulin use, disordered eating, and anxiety disorders. - Psychological problems are associated with suboptimal diabetes self-care (eg, less self-monitoring of glucose levels), diabetes outcomes (eg, HbA_1c_), and psychosocial outcomes (eg, quality of life) [4, 5]. | - Confirmed need for intervention—psychological problems have a significant detrimental impact on the emotional and physical health of adults with type 1 and type 2 diabetes. - Findings informed development of a list of psychological problems (ie, topics for possible *handbook* chapters) and the *handbook* content (eg, each chapter includes a diagram showing the proportion of people with diabetes who typically experience the psychological problem). |
| 1. What do adults with diabetes need, in relation to communication with or support from health professionals for emotional well-being? | - Adults with diabetes regard psychological support as an important component of diabetes care; many would like to talk with their diabetes team about their feelings about living with diabetes [6-8]. - Often those who need to talk (ie, experiencing problems) want to talk [6, 9]. - Psychological problems among people with diabetes often go unidentified and unaddressed [6, 8]. | - Confirmed need for intervention—people with diabetes want emotional support but, typically, are not receiving it. - Findings informed the *handbook* content (eg, it includes information about why attention to the psychological aspects of diabetes is important, from the perspective of people with diabetes). |
| 1. What is the evidence for routine screening for psychological problems in diabetes? | - Mixed evidence and expert opinion about feasibility, efficiency, and effectiveness—some *against* or *uncertain* about the benefits of psychological screening [10-12], but these tended to screen in isolation; several *recommend* it (eg, when implemented well and combined with support and follow-up) [6, 7, 13-16]. - Validated, reliable, and easy-to-use screening tools exist (eg, PAID scale [17] for diabetes distress) and have been implemented successfully in clinical practice ) [6, 7, 13-16]. - When implemented well (eg, collaborative, stepped-care approaches, including follow-up care), routine screening for psychological problems:   - Is acceptable to people with diabetes [6, 7]   - Has positive psychological and physical impacts [14, 15]   - Is cost-effective [18]. | - Confirmed need for intervention—most rigorous studies support routine psychological screening as part of comprehensive diabetes care (including follow-up discussion and treatment and care for identified problems). - Findings informed the *handbook* content (eg, the *handbook* references identified the literature about the evidence for a holistic approach to diabetes care). |
| 1. What do existing diabetes clinical practice guidelines recommend regarding psychological screening and care? | - Australian diabetes guidelines encourage consideration of psychological problems but do not make recommendations to screen or monitor routinely [19, 20].^b^ - Several international guidelines make specific recommendations for routine screening or monitoring of psychological problems [21-23]. | - Confirmed need for intervention—Australian guidelines not aligned with international standards. - Findings informed the *handbook* content (eg, a section about “what the guidelines say” about the psychological aspects of diabetes is included). |
| 1. Does screening for psychological problems occur routinely in diabetes care? | - Guidelines related to screening for psychological problems in people with diabetes are rarely implemented in clinical practice [24]. | - Confirmed need for intervention—recommendations for routine screening exist (internationally) but they are not implemented routinely. - Knowledge gap identified—What are the barriers and enablers to routine screening for psychological problems (See Research Questions 6-7)? - Findings informed the *handbook* content (eg, the *handbook* references identified studies). |
| 1. What barriers affect attention to psychological aspects of diabetes (including routine psychological screening) in diabetes care? | - Routine psychological screening not endorsed in Australian diabetes guidelines [19, 20].^b^ - Health professionals’:   - Lack of, or inadequate access to relevant resources (eg, access to screening tools) [24]   - Lack of skills and training (eg, communication and psychological assessment and care) [24]   - Lack of confidence and feeling uncomfortable with having conversations about the psychological aspects of diabetes [25]   - Perceived lack of time [25]. - Organizational or environmental constraints, including lack of: [24, 25]   - Private spaces (to have sensitive conversations)   - Funding (eg, reimbursement of staff)   - Team or supervisor support   - Team members with psychological training   - Workplace protocols for psychological referrals   - Referral options. - Potential resistance from some patients (eg, refusal or reluctance to participate in screening or discussion) [26]. | - The *handbook* and *toolkit* directly fill the identified resource gap. They:   - Are freely accessible to all diabetes health professionals   - Include copies of the practical tools and resources (validated questionnaires and factsheets for people with diabetes)   - Include user-friendly features, eg, consistent structure, color coding, and chapter summary cards (for busy, time-poor professionals). - The *handbook* includes information to aid health professionals to overcome the lack of skills and confidence barriers to providing support for the emotional aspects of diabetes, eg, the *handbook*:   - Summarizes key clinical guidelines and references peer-reviewed literature related to the psychological aspects of diabetes   - Promotes skill development (for communication and psychological care in diabetes) by providing clinically relevant examples of application   - Includes information about how to weave psychological conversations into routine consultations without compromising the diabetes care agenda   - Provides clear guidance about when to assist and when to refer to another diabetes or mental health professional. - It was not possible to directly influence organizational or environmental constraints, nor perceived lack of time, within the scope of the project. But the *handbook* content acknowledges such barriers and makes suggestions for overcoming them, eg, the *handbook*:   - Makes suggestions for how to set up the consultation room to promote open conversations   - Encourages health professionals to advocate for greater attention to the psychological aspects of diabetes within their workplace (eg, policy development)   - Includes information about referral pathways and making psychological referrals   - Acknowledges that time is a perceived barrier and provides examples of how psychological screening has been successfully implemented in diabetes clinical settings. - It is not the purpose of the *handbook* to make people with diabetes talk about their feelings. It is about creating opportunities for them to talk when they want or need to do so. Thus, the *handbook* makes suggestions for “best practice” ways to introduce and implement psychological screening and discuss the results with the person. - Despite being outside the project remit, we concurrently advocated for inclusion of routine psychological screening in Australian diabetes guidelines.^b^ |
| 1. What facilitates attention to psychological aspects of diabetes (including routine psychological screening) in diabetes care? | - Health professional guidelines recognize the importance of the psychological aspects of diabetes [21-23] - Diabetes health professionals are aware of the need to monitor psychological well-being [25] - Australian and international studies [6. 7. 15] demonstrate that psychological assessment can be implemented successfully in diabetes care settings. | - Confirmed need for intervention—evidence-based guidelines exist, but there is need for guidance and resources to encourage and enable health professionals to implement them. - We incorporated the identified enablers into the resources and their development, for example: - Involvement of health professionals in development and testing, to ensure the final product was relevant, easy to understand, and feasible to implement [27] - The *handbook* summarizes and references relevant evidence - The *toolkit* provides easy access to validated tools and resources. |
| aThis literature review took place at the beginning of the project (2013).  ^b^Psychological screening is now recommended in Australian clinical guidelines for type 2 diabetes (2014, 2016) and in the National Diabetes Strategy (2015) [28-30]. | |  |

# References

1. Smith KJ, Béland M, Clyde M, Gariépy G, Pagé V, Badawi G, et al. Association of diabetes with anxiety: a systematic review and meta-analysis. Journal of Psychosomatic Research. 2013;74:89-99.

2. Li C, Ford ES, Zhao G, Ahluwalia IB, Pearson WS, Mokdad AH. Prevalence and correlates of undiagnosed depression among US adults with diabetes: the Behavioral Risk Factor Surveillance System, 2006. Diabetes Research and Clinical Practice. 2009;83:268-79.

3. Speight J, Browne JL, Holmes-Truscott E, Hendrieckx C, Pouwer F, on behalf of the Diabetes MILES – Australia reference group. Diabetes MILES – Australia 2011 Survey Report. Canberra: 2011.

4. Ciechanowski PS, Katon WJ, Russo JE. Depression and diabetes: impact of depressive symptoms on adherence, function, and costs. Archives of Internal Medicine. 2000;160:3278-85.

5. Fisher L, Hessler DM, Polonsky WH, Mullan J. When is diabetes distress clinically meaningful? Establishing cut points for the diabetes distress scale. Diabetes Care. 2012;35:259-64.

6. Snoek FJ, Kersch NYA, Eldrup E, Harman-Boehm I, Hermanns N, Kokoszka A, et al. Monitoring of Individual Needs in Diabetes (MIND): baseline data from the cross-national Diabetes Attitudes, Wishes, and Needs (DAWN) MIND study. Diabetes Care. 2011;34:601-3.

7. Hendrieckx C, Bowden J, Halliday J, Colman P, Cohen N, Jenkins A, et al. An audit of psychological well-being in adults with type 1 diabetes. Paper presented at: Australian Diabetes Society and Australian Diabetes Educators Society Annual Scientific Meeting Brisbane, Australia2012.

8. Clinical Resource Efficiency Support Team (CREST). Blueprint for Diabetes Care in Northern Ireland in the 21st Century: Report of the User Group Survey. Belfast: 2003.

9. Davies M, Dempster M, Malone A. Do people with diabetes who need to talk want to talk? Diabetic Medicine. 2006;23:917-19.

10. Fleer J, Tovote KA, Keers JC, Links TP, Sanderman R, Coyne JC, et al. Screening for depression and diabetes‐related distress in a diabetes outpatient clinic. Diabetic Medicine. 2013;30:88-94.

11. Burton C, Simpson C, Anderson N. Diagnosis and treatment of depression following routine screening in patients with coronary heart disease or diabetes: a database cohort study. Psychological Medicine. 2013;43:529-37.

12. Pouwer F, Tack CJ, Geelhoed-Duijvestijn PHLM, Bazelmans E, Beekman AT, Heine RJ, et al. Limited effect of screening for depression with written feedback in outpatients with diabetes mellitus: a randomised controlled trial. Diabetologia. 2011;54:741-8.

13. Meeuwissen JAC, Holleman GJM, de Jong FJ, Nuyen J, van der Feltz‐Cornelis CM. Screening and guided self‐help intervention for anxiety and depression in patients with type 2 diabetes. European Diabetes Nursing. 2011;8:47-52a.

14. Pouwer F, Snoek FJ, van der Ploeg HM, Adèr HJ, Heine RJ. Monitoring of psychological well-being in outpatients with diabetes effects on mood, HbA1c, and the patient’s evaluation of the quality of diabetes care: a randomized controlled trial. Diabetes Care. 2001;24:1929-35.

15. Snoek FJ, Kersch NYA, Eldrup E, Harman-Boehm I, Hermanns N, Kokoszka A, et al. Monitoring of Individual Needs in Diabetes (MIND)-2: follow-up data from the cross-national Diabetes Attitudes, Wishes, and Needs (DAWN) MIND study. Diabetes Care. 2012;35:2128-32.

16. Hermanns N, Kulzer B, Krichbaum M, Kubiak T, Haak T. How to screen for depression and emotional problems in patients with diabetes: comparison of screening characteristics of depression questionnaires, measurement of diabetes-specific emotional problems and standard clinical assessment. Diabetologia. 2006;49:469-77.

17. Polonsky WH, Anderson BJ, Lohrer PA, Welch G, Jacobson AM, Aponte JE, et al. Assessment of diabetes-related distress. Diabetes Care. 1995;18:754-60.

18. Pignone MP, Gaynes BN, Rushton JL, Mills Burchell C, Orleans CT, Mulrow CD, et al. Screening for depression in adults: a summary of the evidence for the US Preventive Services Task Force. Annals of Internal Medicine. 2002;136:765-76.

19. Harris P, Mann L, Phillips P, Bolger-Harris H, Webster C. Diabetes Management in General Practice: Guidelines for Type 2 Diabetes: Seventeenth Edition 2011/12. Canberra: 2011.

20. Craig ME, Twigg SM, Donaghue KC, Cheung NW, Cameron FJ, Conn J, et al. National Evidence-Based Clinical Care Guidelines for Type 1 Diabetes in Children, Adolescents and Adults. Canberra: 2011.

21. American Diabetes Association (ADA). Standards of Medical Care in Diabetes. Diabetes Care. 2008;31:S12-54.

22. International Diabetes Federation (IDF). Global Guidelines for Type 2 Diabetes. Belgium: 2012.

23. Scottish Intercollegiate Guidelines Network. Management of Diabetes: A National Guideline. Edinburgh: 2010.

24. Nicholson TRJ, Taylor JP, Gosden C, Trigwell P, Ismail K. National guidelines for psychological care in diabetes: how mindful have we been? Diabetic Medicine. 2009;26:447-50.

25. Mosely K, Aslam A, Speight J. Overcoming barriers to diabetes care: perceived communication issues of healthcare professionals attending a pilot Diabetes UK training programme. Diabetes Research and Clinical Practice. 2010;87:e11-4.

26. Beverly EA, Ganda OP, Ritholz MD, Lee Y, Brooks KM, Lewis-Schroeder NF, et al. Look who’s (not) talking. Diabetes Care. 2012;35:1466-72.

27. Speight J, Conn J, Dunning T, Skinner TC. Diabetes Australia position statement. A new language for diabetes: improving communications with and about people with diabetes. Diabetes Research and Clinical Practice. 2012;97:425-31.

28. Royal Australian College of General Practitioners (RACGP). General Practice Management of Type 2 Diabetes 2014-15. East Melbourne: 2014.

29. Royal Australian College of General Practitioners (RACGP). General Practice Management of Type 2 Diabetes: 2016-18. East Melbourne: 2016.

30. Australian Government Department of Health (DoH). Australian National Diabetes Strategy: 2016-2020. Canberra2015 [4 February 2016]; Available from: www.health.gov.au/internet/main/publishing.nsf/Content/3AF935DA210DA043CA257EFB000D0C03/$File/Australian%20National%20Diabetes%20Strategy%202016-2020.pdf.
